# Viral Infections Confined to Tattoos—A Narrative Review

**DOI:** 10.3390/medicina58030342

**Published:** 2022-02-23

**Authors:** Mircea Tampa, Madalina Irina Mitran, Cristina Iulia Mitran, Clara Matei, Andreea Amuzescu, Alina Andreea Buzatu, Simona Roxana Georgescu

**Affiliations:** 1 Department of Dermatology, ‘Carol Davila’ University of Medicine and Pharmacy, 020021 Bucharest, Romania; tampa_mircea@yahoo.com (M.T.); matei_clara@yahoo.com (C.M.); simonaroxanageorgescu@yahoo.com (S.R.G.); 2 Department of Dermatology, ‘Victor Babes’ Clinical Hospital for Infectious Diseases, 030303 Bucharest, Romania; andreea-paula.amuzescu@rez.umfcd.ro; 3 Department of Microbiology, ‘Carol Davila’ University of Medicine and Pharmacy, 020021 Bucharest, Romania; madalina.irina.mitran@gmail.com; 4 Department of Microbiology, “Cantacuzino” National Medico-Military Institute for Research and Development, 011233 Bucharest, Romania; 5 Department of Communications and Public Relations, Faculty of Letters, University of Bucharest, 010017 Bucharest, Romania

**Keywords:** tattoo, viral infections, molluscum contagiosum, warts, herpes simplex

## Abstract

Since ancient times, people have tattooed their skin for various reasons. In the past, tattoos were associated with low social status; nowadays, tattoos are very popular and are considered a form of art. However, tattoos are associated with various clinical problems, including immune reactions, inflammatory disorders, infections, and even skin cancer. Epidemiological and clinical data of infections on tattoos are scarce. Tattoo-related infections are mostly bacterial; only a few localized viral infections have been reported so far and are caused by molluscum contagiosum virus (MCV), human papillomavirus (HPV), and herpes simplex virus (HSV). In most cases, the lesions were strictly confined to the area of the tattoo. In this review, we have analysed reported cases of viral infections localized on tattoos and discussed the possible mechanisms involved in the occurrence of these infections.

## 1. Introduction

Tattoos represent the introduction of exogenous pigments into the skin to obtain a permanent design [1]. In the last two decades, the prevalence of tattoos has increased significantly [2], ranging from 5 to 40% in adults [3]. Many individuals will have their first tattoo at the age of 16–20 years [4]. Throughout history, tattoos have been performed for various purposes, representing simple decorative elements, a marker of social rank, or the sign of belonging to a certain group [5]. Varying by circumstances, tattoos acquired positive valuations (heralds of distinction and spiritual devotion) and negative overtones (symbols of shame) [6]. Individuals with tattoos may experience stigma, stereotyping, and discrimination [7]. While the temptation of valuing the act of tattooing has been displayed since archaic epochs (Herodotus documents Thracians and Scythians distinguishing tattoos as emblems of nobility, while Greeks and Romans are reported to equate them with stigmata, slavery, and punitive measures) [8], the scientific evidence supporting tattoo correlation with deviant behaviour is demure [9,10].

Tattoos are classified as traumatic, cosmetic, or decorative and can be performed in professional settings or by amateurs [3]. Tattoos can be black or polychrome, but the black colour is predominant; in over 60% of cases, this type of ink is used. They are found in almost all areas of the human body [11]. Histological examination of the tattooed skin revealed the presence of pigment particles in the cytoplasm of various cells, such as fibroblasts and macrophages [11]. Recently, a pigment capture–release–recapture model was described in mice [12]. Macrophages loaded with pigment die after a variable period, the pigment is released, and neighbouring macrophages take it up; in this way, the long-term persistence of tattoos is possible [12,13]. Tattooing represents an important aggression of the skin that leads to the disruption of the epidermal basement membrane and even cell necrosis [1]. Between 1–5% of those who get a tattoo experience tattoo-related skin infections [4]. Infections after tattooing can be caused by endogenous microorganisms, i.e., microorganisms that are part of the normal flora, as a result of altered skin barrier or exogenous microorganisms that enter the skin by inoculation during tattooing [14]. Epidemiological and clinical data regarding infections on tattoos are scarce. The aim of our review is to bring together data on viral infections located on tattoos and to discuss the possible mechanisms involved in the occurrence of these infections. 

## 2. Brief History of Tattoos

Throughout centuries, tattooing has served a deluge of socio-cultural functions, eliciting the perusal of anthropologists, historians, philosophers, sociologists, art critiques, or behavioural economists. Early tattoos employed homemade tools, as indicated in a detailed record of Egyptian practices of the 19th century [15]. In terms of colouring schemes, antique practice examination recounts the prevalence of dark pigments. Nonetheless, brighter colours have also been heavily used, as artefacts of ancient Egyptians and Romans demonstrate a fascination for colours, with red, blue, green, and yellow as prevalent chromatic options [16]. 

While tattooing is an ancient art, academic consensus over the world’s oldest tattoos was only reached in late 2015. Following archaeologists’ perusal through extensive radiocarbon dating, in which an unidentified mummy specimen belonging to the Chinchorro preceramic culture has been compared with Ötzi (a Tyrolean Iceman), the latter obtained the title of the world’s oldest tattoo remains. Discovered in 1991 at the Austrian–Italian border, the specimen had been preserved in ice for more than 5000 years, thus allowing for comprehensive scrutiny of 61 tattoo marks across his body. The body parts (wrist, legs, lower back, torso), alongside the tattoos pattern distribution, hinted at social, symbolic, and therapeutic functions [17]. 

In Ancient Egypt, it was a tradition employed by women, assumed to have consider it to have protective powers, as a remedy during pregnancy. Historical and ethnographic texts register women tattooing as an aid during the reproductive process, as fertility amulet, or for protection in pregnancy and birth phases [18].

Polynesian cultures alone provide a full-bodied universe for examination, with heavily infused cultural expressions and meticulous geometries. Tattoos compensated for the absence of a form of written culture, thus acting as ways of signaling ancestry, identity narratives, abilities, awards, social status, personal accomplishments, and collective experiences [19]. Thereupon, the etymology of the modern term “tattoo” stems from this cultural space, where tatatau or tattau means to hit or to strike [16]. 

The configuration of modern Western tattoos was shaped by colonialism, seafaring, electric tattoo machine technology, health regulations, and the consumer marketplace [20]. Academic papers and public discourse alike mention Captain James Cook as the bearer who introduced cross-cultural tattooing practices into the continent, following his 1770s Tahiti, New Zealand and the Pacific region explorations. Nonetheless, recent scrutiny of primary sources indicates the dissemination value of these voyages, rather than the acquisition of novel cultural contacts, in an attempt to demystify the absence of recorded European tattoo practices before the adventures of Captain Cook [21].

The 18th and 19th century in Europe and the U.S.A. present tattoos as constitutive characteristics of marginalized communities (financially destitute or socially corrupted groups). The late 19th century captivated cultural elites into pursuing tattoos as an endeavour to signal exoticism or distinction, a short-lived frivolity [22] that ended with the democratization of the practice in 1891. Both a Navy member and a convict, Irish American Samuel O’Reilly acquired the patent number 464,801 for the first tattooing machine [23].

These were centuries dominated by the use of contaminated ink and needles; a 19th century medical report on a syphilis outbreak in the outskirts of a naval barrack reveals the use of saliva during the process [24]. A tattoo artist of the 1950s explains this misunderstanding by referencing an article in the *Journal of the American Medical Association*; imported folklore misinterpreted tattooing risk factors with a syphilis cure, augmenting the confusion between the adverse and therapeutic effects of mercury-based pigments [25]. 

The middle of the 20th century is acknowledged as a period of tattoo renaissance, with the 1960’s counterculture relocating conventions through shifts in civil rights, public rhetoric, visual culture, corporality, subcultures, peripheral group perception, and the representation of alterity [26]. 

Aided by the systemic shift from trade to art [9], tattoos of the 21st century are crafted in schools for tattoo artists and deliberated within aesthetic, art history, and cultural theories. The commodification of the praxis has been, in the realm of popular culture, heavily augmented by the mainstream discourse and digital content [20].

## 3. Materials and Methods

We performed a narrative review by interrogating the PubMed and Google Scholar databases with the following combinations of terms, “tattoo and infection”, “tattoo and molluscum contagiosum”, “tattoo and warts”, “tattoo and verruca”, and “tattoo and herpes simplex”. We have selected the articles presenting cases of viral infections confined to tattoos. We have excluded the cases reporting systemic infections after tattooing. After applying these criteria, we have identified 14 case reports of molluscum contagiosum virus (MCV) infection; 18 case reports, 2 reports of two cases, and 2 case series of warts; and 4 case reports of herpes simplex virus (HSV) infection.

## 4. Viral Infections Localized on Tattoos

The proliferation of tattoo artists, studios, and parlours, the emergence of technological innovations such as ink variety, the rise in media reports fed insignificant complementary changes in regulations for sterilization process, and hygiene standard practices [27]. Whereas tattoo shops have formalized safety and sanitation measures, appropriate institutional control on the practice is lax, since tattooing is considered a beautification activity, except for two requisite resolutions (ResAp in 2003 and revised ResAp in 2008) from the Council of Europe [28]. When measured against other cultural production modes, tattooing is one of the most extensively regulated processes [29]. 

Amid deliberation on the formal placement and regulation of the praxis, medical research continues to gather a body of data that can offer further insight into compliant technologies, potential risks, and adverse reactions. The past decade generated abundant clinical reports of tattoo complications, investigating reactions based on the quality of the pigments, the techniques, after-care exposure, body parts, and even on ink colours [28].

The main complications after tattoo placement include immunologic reactions (allergic dermatitis, immunohypersensitivity, etc.), inflammatory skin disorders (psoriasis, lichen planus, pseudolymphoma, etc.), infections (viral, bacterial and fungal), and neoplasms (lymphoma, melanoma, basal cell carcinoma, etc.) [1,30,31]. Infections may be localized or systemic; most are bacterial. Serious viral infections include hepatitis B and C and human immunodeficiency virus (HIV) infection. Localized skin infections are rare and are caused by MCV, human papillomavirus (HPV), and HSV [32,33,34].

### 4.1. Molluscum Contagiosum

Molluscum contagiosum (MC) is a self-limited cutaneous viral infection that affects mainly children, sexually active adults, and immunosuppressed individuals [35,36]. MC is caused by MCV, a double-stranded DNA virus that belongs to the Poxviridae family. MCV has four different genotypes, with MCV-1 being the most common genotype (75 to 96% of cases) [37]. MCV infects the epidermis and replicates in the cytoplasm of cells, but does not exceed the basement membrane [35,38]. The incubation period varies between 2 weeks and 6 months [39]. The replication of the virus in keratinocytes promotes the formation of characteristic eosinophilic cytoplasmic inclusions (Henderson–Paterson bodies) [37]. Clinically, MC is characterized by firm, pearly white or pink, dome-shaped, centrally umbilicated papules that range in size from 1 to 5 mm in diameter. In most cases the lesions are asymptomatic; pruritus is uncommon. The lesions may appear grouped or may adopt a linear arrangement in the case of autoinoculation [36]. MCV infects almost exclusively the skin and rarely the mucous membranes [38].

Table 1 summarizes the cases of MC on tattoos reported in the medical literature. The cases have been reported mainly in young males. In no cases, immunosuppression was specified. The ages of the patients ranged from 16 to 64 years.

In 1982, Foulds reported the first case of MC on a tattoo performed using carbon, scarlet lake, and chlorinated copper pigments. The authors described the case of a 20-year-old man who presented with seven lesions of MC confined to the area of the tattoo performed with carbon pigment. The lesions healed spontaneously after 6 months [40]. However, it seems that the first case was described by Bergh in 1903 [54].

**Figure 1:**
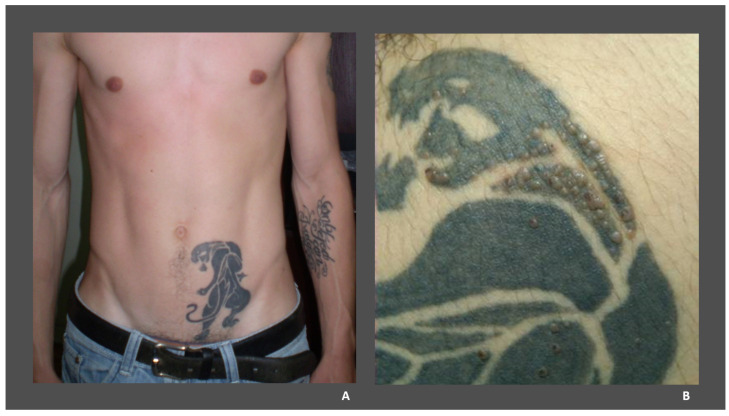
(**A**) A 32-year-old man with molluscum contagiosum on a black tattoo located on his left abdominal flank; (**B**) molluscum contagiosum papules strictly confined to the tattooed skin (images from our clinic, previously published in Tampa et al., 2012 [49]).

Most cases reported in the literature show that MC lesions are confined to the tattooed skin, with no involvement of the surrounding skin (Figure 1). Ruiz-Villaverde et al. have reported the case of a 23-year-old immunocompetent male, who presented with MC lesions both on his black tattoo and on the adjacent skin [50]. Furthermore, the analysis of the reported cases showed that black ink was used in most cases. The latency period between tattooing and the appearance of MC lesions ranged from 2 weeks to 5 months. 

### 4.2. Warts

HPV is a DNA virus with tropism for the skin and mucous membranes that can be classified into five genera (Alpha, Beta, Gamma, Mu, and Nu). Depending on the oncogenic risk, HPV types are divided into high-risk types associated with malignant lesions and low-risk types that cause benign lesions, in most cases asymptomatic, with limited evolution. Usually, the host’s immune response induces the viral clearance [55,56,57,58]. Warts are the most common skin manifestations of HPV infection. According to their morphology and anatomical site, several subtypes of warts (common, plantar, and flat warts) have been described. Common warts, also known as verruca vulgaris, are the most frequently seen and classically appear as hyperkeratotic papules [59]. Flat warts (verruca plana) are less common and manifest as flat-topped papules. Generally, the incubation period of warts varies between 1 and 6 months [60].

We have analyzed 22 case reports of warts on tattoos (Table 2). It seems that Fox was the first to describe warts on a tattoo in 1864; however, at that time their viral etiology was unknown [61]. 

**Figure 2:**
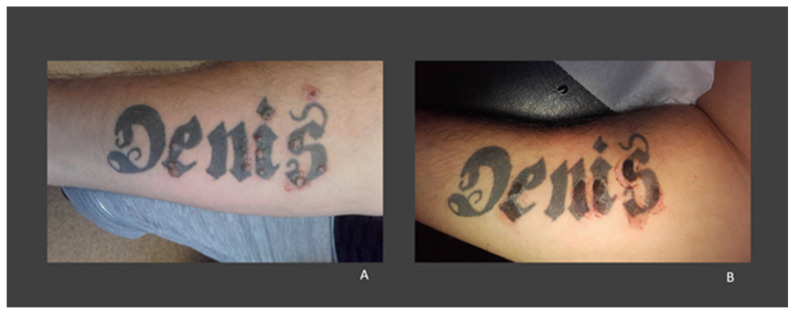
(**A**) A 30-year-old man with warts on a tattoo with black pigment on his left forearm. (**B**) The aspect of the tattoo after curettage and electrosurgery of the lesions (images from our clinic, previously published in Georgescu et al., 2017 [76]).

The age of the patients ranged from 17 to 66 years, but most cases were reported among young men. The period from when the tattoo was obtained and the appearance of warts varied between 2 months and over 20 years. In most cases, the warts were restricted to the tattooed skin and were preferentially located on black tattoos (Figure 2). Veasey et al. reported two cases: in one case, the patient was immunocompetent; in the second, the patient was infected with HIV (the only case identified in an immunocompromised patient). In the first case, the warts were located only on the tattooed skin, in contrast to the second case, in which they were also observed on healthy skin [80]. However, in the case of an immunocompetent woman, reported by Jung et al., warts initially appeared on the tattooed skin (eyebrow), but later spread to her face [70]. In all other cases, the warts were located exclusively on the tattooed area. In most cases, HPV testing was not performed; the diagnosis was made from the clinical and histopathological aspects. When HPV testing was performed, HPV 27 [71], HPV 47 [74], and HPV6B [66] types were identified.

Kluger et al. analysed eight cases of warts on tattoos (not included in Table 2). In seven of the eight cases, warts developed on black tattoos. The latency period varied between 6 and 18 months. One patient stated that the warts were present prior to the tattoo placement [82]. In line with this, Ramey et al. analysed 5 cases of warts on tattoos (not included in Table 2) and identified 181 lesions that were located mainly on the areas with black ink [83]. Warts were seven times more likely to be localized on the skin where the black ink was used compared to coloured ink and normal skin [83]. 

### 4.3. Herpes Simplex

HSV-1 and HSV-2 are DNA viruses, members of the *Herpesviridae* family, with a short reproductive cycle, but with the ability to establish latency in the sensory ganglia and may recur in response to various stimuli to produce disease. Classically, HSV-1 causes orofacial lesions and HSV-2 genital lesions [84]. However, today it is known that there is significant overlap between the sites of HSV infection [85]. HSV-1 infections are more common; over 60% of individuals have antibodies against HSV-1, but only 10% of individuals against HSV-2 [86]. In many cases, the infection is asymptomatic and manifests, depending on the affected area, as oral ulcerations, vesicles, or genital lesions [87].

Marshall et al. reported the first case of herpes simplex that appeared as a complication of a tattoo in a 30-year-old man. Bacteriological examination revealed a co-infection with methicillin-susceptible Staphylococcus aureus. The patient had no history of herpes simplex and was treated with intravenous flucloxacillin and oral famciclovir, and the outcome was favourable. Marshall et al. proposed the term herpes compuctorum to describe cutaneous HSV infection that complicates tattoos [88]. In the medical literature, four cases of herpes simplex were reported on tattoos, with three identified in females (Table 3). The age of the patients ranged from 30 to 48 years. Two of the patients had a positive history of HSV infection. Unlike MC and warts, the latency period was very short as 2–3 days.

## 5. Summary of the Characteristics of Viral Infections Confined to Tattoos

In terms of age, the highest mean age was observed among patients with HSV infection. In patients with MC and warts, the values were similar. The small number of patients with HSV infection should be considered. In the group of patients with HSV infection, the number of women was higher, unlike the other two groups in which men were predominant. With regard to the latency period, the differences between groups were significant, ranging from years to days (Table 4). In all cases, the lesions predominantly involved the dark area of the tattoo.

## 6. Potential Mechanisms Involved in the Occurrence of Viral Infections on Tattoos

Over time, several theories have been postulated about the mechanisms of viral infections located on tattoos. The microorganisms can be inoculated through contaminated instruments, ink, or artist saliva, or in some cases, the tattoo is placed on already infected skin [79,83,92]. The procedure itself can lead to the activation of the virus in the case of previously infected people [91]. In addition, overinfection of a freshly healing wound may be possible [89]. 

Ramey et al. suggested that the use of contaminated ink is unlikely to be the source of infection for warts given that HPV is a fastidious virus and has a low chance of surviving in ink [83]. Moreover, Krecke et al. identified HPV-47 in tattoo-associated flat warts. HPV-47 is a member of the Beta genus, whose reservoir is the hair follicle. It should be noted that the act of tattooing could promote the release of the virus [74]. Additionally, Cortes et al. highlighted that MCV cannot survive in the dermis and therefore direct inoculation is less likely [52]. 

Immunity plays an important role in the development and evolution of infections. Human leukocyte antigens (HLAs), which represent genetic markers with important functions in the immune response to microorganisms, vary considerably among individuals. For example, a recent study showed that the HLA-A * 02, HLA-DQA1 * 03:01, and DQA1 * 05:01 genes are more prevalent in patients with warts than in healthy subjects [93]. Another theory that tries to explain the mechanism of the occurrence of viral infections on tattoos is the theory of the immunocompromised district, which refers to a local alteration of the skin’s immune system [94]. Ruocco et al. stated that skin areas that have suffered burns, irradiation, or trauma should be considered ‘loci minoris resistentiae’, i.e., areas likely to develop subsequent skin diseases [94]. This theory is supported by the appearance of several infectious dermatological diseases [95] and non-infectious dermatological diseases (pemphigus [96], basal cell carcinoma [97], fixed post drug rash [98]) on damaged skin. The tattoo pigment reaches the dermis, where it can stimulate an abnormal immune response that contributes to local immunosuppression. In addition, the decomposed pigment in the lymph nodes may be responsible for the immunosuppressed areas located proximal to the tattoo [52]. The long periods between the time of tattoo placement and the development of the infection suggest that microorganisms contracted incidentally, replicated preferentially within the immunocompromised area where the local immune response was altered by ink [99]. 

The localization of the viral infections predominantly within black tattoo areas can be explained by the fact that the black ink causes the alteration of local humoral and cellular immunity [43]. Since 1994, it has been suggested that black dye induces a local immunosuppressive effect [66]. Black inks are produced by imperfect combustion and contain high amounts of polycyclic aromatic hydrocarbons (PAHs) [100,101]. After exposure to ultraviolet (UV) light, PAHs lead to the generation of reactive oxygen species (ROS) that exert harmful effects on cells, causing the peroxidation of membrane lipids and proteins and consequent deterioration of the skin barrier [102,103,104,105]. In addition, black ink contains nanoparticles, unlike coloured ink, which contains larger particles, and the nanoparticles stimulate the production of ROS to a greater extent [103,106]. It should be noted that PAHs do not remain localized only in the dermis. PAHs are also transported to other organs [107].

Ultraviolets are involved in the pathogenesis of many skin diseases [108,109]. UV radiation may have a role in the activation of HPV. UVB radiation promotes HPV activity in keratinocytes, which could also explain the variation in the latency period [110]. Brajac et al. reported the case of a 32-year-old man who developed multiple warts on a tattoo performed 2.5 years previously. The warts appeared two weeks after an acute sunburn involving tattooed and normal skin, but the warts were confined to the tattooed area, suggesting that the virus was probably inoculated at the time of tattooing and remained latent, and the UV light acted as a trigger inducing its activation [68]. Rho et al. highlighted the onset of flat warts on the Q-switched laser-assisted tattoo removal site in a 23-year-old patient, 3 weeks after the procedure [111]. The authors suggested that HPV was probably latent within tattooed skin, and that the treatment caused the alteration of the epidermal barrier that allowed the virus to spread. This observation is based on the report of Amella et al. who, using an animal model, showed that HPV activation could occur after mild skin irritation [112].

## 7. Conclusions

Viral infections localized to tattoos have been reported mainly in young, immunocompetent adults, predominantly males. The lesions were confined to the tattooed area, rarely affecting the surrounding skin. Moreover, the lesions involved preferentially black tattoos. The mechanism of infection remains unclear. The increased latency periods between tattoo placement and lesion appearance support the hypothesis of altered local immunity; however, the use of contaminated ink or instruments cannot be ruled out. Tattoos are becoming more common in modern society and it is important to know all the possible complications, even the rarest.

## Figures and Tables

**Table 1:** Epidemiological and clinical characteristics of the reported cases of molluscum contagiosum on tattoos.

Sex	Age (Years)	Tattoo Localisation	Ink Colour	Latency Period	Reference
male	20	left arm	polychrome- carbon pigment *	3 months	Foulds (1982) [40]
female	20	left forearm	black	3 weeks	Salmaso et al. (2001) [41]
male	20	left calf	polychrome-brownish-grey ink *	5 months	Perez Gala et al. (2006) [42]
male	59	chest	monochromatic	3 months	Kluger et al. (2007) [43]
male	24	right lumbar region	black	2 weeks	Panasiti et al. (2008) [44]
male	36	right arm	black	3 weeks	Perez-Barrio et al. (2009) [45]
male	30	arm	black	20 days	De Giorgi et al. (2010) [46]
male	22	back	black	4 weeks	Molina et al. (2011) [47]
male	33	right upper arm	polychrome	5 months	Grillo et al. (2012) [48]
male	32	left abdominal flank	black	3 weeks	Tampa et al. (2012) [49]
male	23	right abdominal flank	black	N/A	Ruiz-Villaverde et al. (2013) [50]
male	33	right arm	black	3 months	Blasco Morente et al. (2016) [51]
female	16	posterior thorax	blue	N/A	Cortes et al. (2017) [52]
female	64	right eyebrow	dark	1 month	Marcelino et al. (2021) [53]

* Colour of the ink on which the lesions appeared. N/A—not available.

**Table 2:** Epidemiological and clinical characteristics of the reported cases of warts on tattoos.

Sex	Age (Years)	Tattoo Localisation	Ink Colour	Latency Period	Reference
male	21	left arm	black	soon after tattooing	Watkins (1961) [62]
male	24	left scapula	black	2 years	Young et al. (1979) [63]
male	21	N/A	black	6 months	Baxter et al. (1993) [64]
male	27	right scapular area	dark blue	8 years	Ragland et al. (1994) [65]
male	33	right upper arm	black	8 years	Miller et al. (1994) [66]
male	29	left arm	polychrome-dark blue *	8 years	Trefzer et al. (2004) [67]
male	32	back (left scapular area) after sunburn	dark blue	2.5 years	Brajac et al. (2005) [68]
female	17	left leg	dark blue and red-dark blue *	3 months	Saez et al. (2006) [69]
female	39	face	dark	1 year	Jung et al. (2009) [70]
female	31	right ankle	polychrome	2 years	Wanat et al. (2014) [71]
male	25	the back of the left hand	polychrome	N/A	Navarro-Vidal et al. (2015) [72]
male	24	right arm	polychrome-dark, red *	4.5 years	Fania et al. (2017) [73]
male	35	left arm	polychrome-dark *	3.6 years	Fania et al. (2017) [73]
male	47	forearm	black	12 years	Krecke et al. (2017) [74]
female	66	both eyebrows	dark grey/black	8 years	Nemer et al. (2018) [75]
male	30	left forearm	black	3 months	Georgescu et al. (2017) [76]
male	36	right forearm	black	more than 20 years	Kirchhof et al. (2019) [77]
male	25	right upper arm	dark blue	3 months	Yuan (2019) [78]
female	27	left leg	black	2 years	Chen et al. (2020) [79]
male	39	left calf	polychrome	1 year	Veasey et al. (2020) [80]
male	33	left upper limb	polychrome	6 years	Veasey et al. (2020) [80]
female	44	left lower leg	polychrome-black *, red	26 years	Cohen(2021) [81]

* Colour of the ink on which the lesions appeared. N/A—not available.

**Table 3:** Epidemiological and clinical characteristics of the reported cases of herpes simplex on tattoos.

Sex	Age (Years)	Tattoo Localisation	Ink Colour	Latency Period	Reference
male	30	left arm	black	3 days	Marshall et al. (2007) [88]
female	31	right flank	black	3 days	Kluger et al. (2017) [89]
female	48	lips	red	2 days	AlQuorain et al. (2017) [90]
female	46	lips	red	2 days	Begolli Gerqari et al. (2018) [91]

**Table 4:** Demographic characteristics of the reported cases.

Parameter	Molluscum Contagiosum	Warts	Herpes Simplex
Age (mean ± SD)	30.85 ± 14.38 years	32.04 ± 10.7 years	38.75 ± 9.56 years
Male:Female ratio	3.6:1	2.6:1	0.3:1
Latency period (mean ± SD)	2.04 ± 1.69 months	5.93 ± 6.77 years	2.5 ± 0.56 days
